# Articulation-Function-Associated Cortical Developmental Changes in Patients with Cleft Lip and Palate

**DOI:** 10.3390/brainsci13040550

**Published:** 2023-03-25

**Authors:** Wenjing Zhang, Cui Zhao, Liwei Sun, Xintao Yang, Linrui Yang, Ying Liang, Xu Zhang, Xiaoxia Du, Renji Chen, Chunlin Li

**Affiliations:** 1 Beijing Stomatological Hospital, Capital Medical University, Beijing 100050, China; 2 School of Stomatology, Capital Medical University, Beijing 100050, China; 3 School of Artificial Intelligence, Beijing University of Posts and Telecommunications, Beijing 100876, China; 4 Beijing Key Laboratory of Fundamental Research on Biomechanics in Clinical Application, School of Biomedical Engineering, Capital Medical University, No. 10 Xitoutiao, You An Men Wai, Beijing 100069, China; 5 China Rehabilitation Research Center, Capital Medical University, Beijing 100068, China

**Keywords:** cleft lip and palate, articulation disorder, cortical development, mediation effect

## Abstract

Cleft lip and palate (CLP) is one of the most common craniofacial malformations. Overall, 40–80% of CLP patients have varying degrees of articulation problems after palatoplasty. Previous studies revealed abnormal articulation-related brain function in CLP patients. However, the association between articulation disorders and cortical structure development in CLP patients remains unclear. Twenty-six CLP adolescents (aged 5–14 years; mean 8.88 years; female/male 8/18), twenty-three CLP adults (aged 18–35 years; mean 23.35 years; female/male 6/17), thirty-seven healthy adolescents (aged 5–16 years; mean 9.89 years; female/male 5/16), and twenty-two healthy adults (aged 19–37 years; mean 24.41 years; female/male 19/37) took part in the experiment. The current study aims to investigate developmental changes in cortical structures in CLP patients with articulation disorders using both structural and functional magnetic resonance imaging (MRI). Our results reveal the distinct distribution of abnormal cortical structures in adolescent and adult CLP patients. We also found that the developmental pattern of cortical structures in CLP patients differed from the pattern in healthy controls (delayed cortical development in the left lingual gyrus (t = 4.02, cluster-wise *p* < 0.05), inferior temporal cortex (z = −4.36, cluster-wise *p* < 0.05) and right precentral cortex (t = 4.19, cluster-wise *p* < 0.05)). Mediation analysis identified the cortical thickness of the left pericalcarine cortex as the mediator between age and articulation function (partial mediation effect (a*b = −0.48), 95% confident interval (−0.75, −0.26)). In conclusion, our results demonstrate an abnormal developmental pattern of cortical structures in CLP patients, which is directly related to their articulation disorders.

## 1. Introduction

Cleft lip and palate (CLP) is one of the most frequent craniofacial congenital defects [1]. In Asia and North America, the prevalence of CLP is as high as 1 in 700 [1,2], whereas the prevalence in China is approximately 1.95 per 1000. After palatoplasty, 40% to 80% of children experience various speech problems [3,4,5], with hypernasality and glottal stop as the predominant compensatory articulation characteristics [6]. Improving and restoring speech function in CLP populations is the primary objective of surgical treatment for CLP.

Neuroimaging studies have shown significant cortical structure abnormalities among individuals with CLP. Previous research has found altered cortical structures in adults with CLP, primarily in the frontal lobes, temporal lobes, parietal lobes, occipital lobes, midline brain, posterior cerebrum, cerebellum, orbitofrontal cortex, superior temporal plane, cerebral poles and corpus callosum [7,8,9,10,11,12,13]. In adults with CLP, these abnormal cortical structures are directly associated with cognitive processes such as language, intelligence quotient (IQ) and social function. In CLP adults, the abnormally increased superior temporal plane volume was directly related to a lower IQ [9,11]. The decreased volume of the orbitofrontal cortex in adult males with CLP has been linked to impaired social performance [13]. After receiving speech therapy, CLP adults demonstrated structural plasticity in brain regions responsible for speech function (left inferior parietal cortex and right supramarginal gyrus), auditory function (right temporal pole and right inferior temporal gyrus), articulatory planning and execution (right supplementary motor area) [10]. Children and adolescents with CLP are in the developmental period of brain maturation, and their cortical structure abnormalities are partially distinct from those of CLP adults. The abnormal cortical structure consists primarily of the bilateral medial frontal cortex, the supplementary motor area, the middle cingulate and the gray matter of the dorsomedial nuclei of the bilateral thalamus, indicating that the likelihood of abnormal cognitive function in children with CLP is higher than that of normal children [14]. CLP boys have a smaller auditory cortex and ventral frontal cortex than normal boys, which may explain their aberrant social conduct [9,11].

Articulation disorder is one of the developmental motor-speech disordersthat can persist into adulthood [15]. Previous studies have shown a correlation between articulation function and brain activation in CLP patients [16,17]. CLP subjects recovered to a normal level of articulation function after speech training, while their language-related brain function was improved [18]. The changes in gray matter (GM) can reflect language skills; for instance, impaired readers have reduced GM in the ventral and dorsal regions of the reading network [19]. The language ability improves as the brain matures [19]. Previous research has shown that language-related brain areas are widespread and localized in both hemispheres during childhood [20]. However, language is lateralized to the left hemisphere in most neurologically healthy adults [21]. Compared to preschoolers, adults have more developed white matter in the arcuate fasciculus and widely distributed language-associated cortical regions, providing morphological evidence for changes in language development [22]. Children acquire substantially fewer associations than adults do within semantic networks [23,24]. Meanwhile, GM might also reveal articulation function in CLP people. It was reported that CLP children with lower levels of articulation have reduced GM volume in language-related regions, such as the left inferior temporal gyrus, right inferior parietal limbic gyrus and left superior cerebellar [25]. GM volume changes in the frontal, temporal and parietal lobes may be uniquely associated with speech disorders in children with CLP. However, to date, no studies have attempted to illustrate the developmental pattern of the brain structure in CLP patients and to relate their specific cortical development pattern to CLP-related speech disorders.

Healthy adolescents and adults have different patterns of cortical structural changes in the brain [26]; moreover, the development of function ability is always associated with the generation of cortical structure changes in function-related brain regions [27]. In the current study, we hypothesized that CLP subjects with articulation disorder have different patterns of the cortical structure and functional changes from adolescence to adulthood. In the current study, we aimed to investigate developmental changes in cortical structures in CLP patients with articulation disorders using both structural and functional magnetic resonance imaging (MRI). We compared the difference in GM volumetrics, cortical thickness and cortical areas between CLP and healthy participants during development. Furthermore, we also detected the mediation effect of the cortical structure between age and articulation function in CLP. We found an abnormal pattern of cortical development in CLP individuals, which is directly related to their articulation disorders.

## 2. Materials and Methods

### 2.1. Participants

A total of 120 participants were recruited from the Beijing Stomatological Hospital affiliated with Capital Medical University; CLP patients were recruited from the CLP Treatment Center of Beijing Stomatological Hospital, and healthy control participants were recruited from volunteers through advertisements in schools and hospitals from January 2017 to January 2021. During the experiment, a total of 12 participants were excluded from screening. Six CLP adolescents displayed head movements during MRI scanning, three CLP adults could not undergo MRI scanning due to their teeth having been repaired with metal material, two healthy adolescents gave no reason and one healthy adult could not stand the noise of MRI scanning. Finally, 108 participants participated in the current study, including 49 patients with CLP (26 children and adolescents and 23 adults) and 59 age- and gender-matched healthy controls. All participants were native Chinese speakers with a normal intelligence quotient (IQ ≥ 90) measured with the Wechsler Intelligence Scale, and all were right-handed. All participants were free from any neurological disease or psychiatric disorder. CLP patients underwent palate repairing surgery about 1 year before the recruitment, and they all completed the assessment of articulation function. CLP subjects participated in a Chinese articulation test, and a test score lower than 70 out of 100 indicated moderate or severe levels of articulation disorder. The critical exclusion criteria for the present study were neurological or psychiatric disorders and contraindications of fMRI. (More detailed inclusion and exclusion criteria can be found in the Tables S1 and S2). This study was approved by the Medical Research Ethics Committee of Capital Medical University, China (CMUSH-IRB-KJ-PJ-2019-04), and written informed consent was obtained from each participant or their parents.

### 2.2. MRI Acquisition and Preprocessing

Both T1-weighted (T1w) structural data and resting-state functional MRI (rs-fMRI) data were collected for each participant using a 3.0 T Philips Achieva MRI scanner with a 32-channel head coil in Beijing Chaoyang Integrative Medicine Emergency Medical Center. High-resolution T1w MRI images were acquired using the axial turbo field echo sequence with the following parameters: repeat time (TR) = 8.2 ms, echo time (TE) = 8.2 ms, flip angel (FA) = 8°, field of view (FOV) = 240 × 240 mm^2^, acquisition matrix = 240 × 240, voxel size = 1 mm^3^. Resting-state functional MRI images were acquired using the axial field-echo echo-planar-imaging sequence with the following parameters: TR/TE = 2/0.035 ms, FA = 90°, FOV = 80 × 80 mm^2^, acquisition matrix = 76 × 76, 240 volumes, voxel size = 2.88 × 2.88 × 4 mm^3^.

### 2.3. Volumetric and Cortical Reconstruction for T1w Imaging Data

Before preprocessing, all T1w and resting-state functional imaging data were visually checked for the same orientation. Volumetric segmentation and cortical reconstruction of T1w data were performed using the Computational Anatomy Toolbox (CAT12 version 12.8; https://neuro-jena.github.io/cat/, accessed on 31 January 2023) implemented in Statistical Parametric Mapping (SPM12; www.fil.ion.ucl.ac.uk/spm/software/spm12/, accessed on 13 January 2020) and FreeSurfer toolbox (version 7.3.2; https://surfer.nmr.mgh.harvard.edu/, accessed on 8 August 2022). In the voxel-based morphometry (VBM) analysis, the T1w image of each participant was segmented and normalized to the standard Montreal Neurological Institute (MNI) space using the Diffeomorphic Anatomical Registration Through Exponentiated Lie Algebra (DARTEL) algorithm [28]. The modulated normalized grey matter (GM) maps were smoothed with a 6 mm full-width-at-half-maximum (FWHM) Gaussian kernel. Cortical reconstruction analysis was performed using the FreeSurfer toolbox. The automatic processing procedure included skull stripping, tissue segmentation, white matter and pial surfaces reconstruction, surface inflation, registration to a spherical atlas, parcellation and extraction of surface-based indices, including the cortical surface, cortical surface area and mean curvature. All cortical indices were normalized to a template image called fsaverage and smoothed with a 15 mm FWHM Gaussian kernel. Each processing step was visually inspected.

### 2.4. Seed-Based Functional Connectivity Analysis for Cortical Regions with a Significant Interaction Effect

The rs-fMRI data were analyzed using CONN (version 20b; https://web.conn-toolbox.org/, accessed on 1 December 2020), a functional connectivity toolbox [29]. In the image quality checking before pre-processing, functional data of two CLP participants (one adolescent and one adult) were excluded due to serious artifacts caused by head motion. One hundred and six participants were included in the seed-based functional connectivity analysis. The preprocessing procedure of rs-fMRI included functional realignment and unwarping of the first scan of each session, slice-timing correction, outlier identification, tissue segmentation and normalization to MNI space and spatial smoothing with a Gaussian kernel of 8 mm FWHM. Global mean signals, noise components from cerebral white matter and cerebrospinal areas and 12 potential noise components from estimated subject-motion parameters were regressed out. This was followed by temporal bandpass filtering (0.008–0.0.09 Hz). Each region exhibiting significant group differences or interaction effects in the comparison of cortical structural indices was defined as a seed, and the seed-based connectivity maps were created for each participant using Fisher-transformed bivariate correlation coefficients between mean regional timeseries of the self-defined seed and each individual voxel timeseries.

### 2.5. Statistical Analysis

Demographic characteristics were compared between CLP and healthy control (HC) participants using an independent sample *t*-test for age and a chi-square test for gender. We also investigated demographic group differences between CLP and HC according to different ages. Significance was set at *p* < 0.05.

Statistical analyses of voxel- or vertex-wise cortical structural maps were conducted with a general linear model (GLM). Group differences were assessed using an independent *t*-test, with age and gender controlled as covariances. To verify the presence of an interaction between age and group on cortical structure, we further compared whether there is a significant difference between the group–age slopes using GLM, and gender was entered as a covariate. For the seed-based functional connectivity maps, group differences were also assessed using GML, with age and gender being controlled. Statistical significance of voxel-wise cortical volumetric analysis was set at voxel-wise *p* < 0.001 and cluster-wise *p* < 0.05 with Gaussian theory field (GRF) correction for multiple comparisons. Vertex-wise statistical analysis of cortical indices was deemed significant at vertex-wise *p* < 0.001 and cluster-wise *p* < 0.05 using Monte Carlo simulation with 5000 iterations. The statistical significance of seed-based functional connectivity was set at cluster-wase family-wise error (FWE)-corrected *p* < 0.05.

To further investigate the association among cortical structure, age and language function in CLP, we conducted mediation analyses by means of structural equation modelling using the R package bruceR (version 0.8.9; https://CRAN.R-project.org/package=bruceR, accessed on 11 August 2022). Mediation analyses were carried out for cortical structural indices of regions exhibiting significant group differences or interaction effects or regional means extracted based on the parcellation of the Desikan–Killiany Atlas. Gender was regressed out as a covariance in the mediation analyses, with a 10,000 bias-corrected bootstrap sample for significance testing. When the bootstrapped confidence interval does not contain zero with 95% confidence, a significant mediation effect is indicated. The total intracranial volume (TIV) of each participant was controlled as a covariance in statistical analyses of the GM volumetric index.

## 3. Results

### 3.1. Demographics

A total of 108 participants were enrolled in this study, with complete structural and functional MRI scans collected, including 49 CLP participants and 59 age- and gender-matched healthy controls (Table 1). A total of 63 children and adolescents, which we called “adolescents” in our study, were included, including 26 CLP adolescents (CLP_ado) (8 female, mean age = 8.88 (range: 5–14)) and 37 healthy adolescents (HC_Ado) (10 female, mean age = 9.89 (range: 5–16)). A total of 45 adults were enrolled, including 23 CLP adults (CLP_Adu) (6 female, mean age = 23.35 (range: 18–35)) and 22 healthy adults (14 female, mean age = 24.41 (range: 19–37)). There was no significant group difference in age and gender between CLP and HC overall. When compared for different ages (adolescents and adults), no significant difference was found in the adolescent groups, but the age of HC_Adu was significantly higher than that of CLP_Adu participants (*p* = 0.011). Participants with CLP also completed the measurement of articulation function, resulting in a mean score = 46.82 ± 23.92 (0–93) (presented as the mean ± standard deviation (range)), specifically in CLP_Ado (44.83 ± 25.73 (0–93)) and in CLP_Adu (51.00 ± 23.57 (13–93)).

### 3.2. The Differences in Gray Matter Volume between Cleft Lip with Palate (CLP) Participants and Healthy Controls across Development

**Figure 1:**
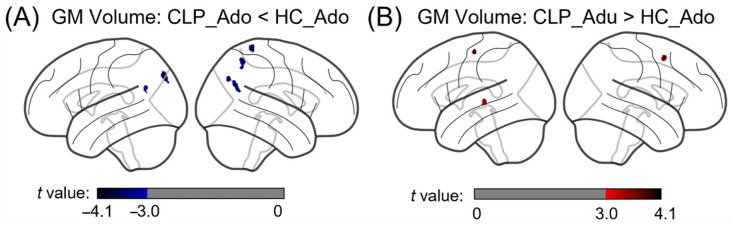
Grey matter (GM) volume with significant group differences between CLP and HC. (**A**) Anatomical locations of clusters with significant group differences, the GM volume of which was significantly decreased in CLP adolescents compared to HC adolescents in the volume-based morphometry analyses; (**B**) anatomical locations of clusters with significant group differences between CLP and HC adults—the volume within these regions was significantly increased in CLP adults compared to HC adults. Gender, age and total intracranial volume (TIV) were regressed out as covariates, and significance was set at cluster-wise Gaussian theory field (GRF)-corrected *p* < 0.05. CLP_Adu = adults with cleft and palate (CLP), HC_Adu = healthy control adults, CLP_Ado = children and adolescents with CLP, HC_Ado = healthy children and adolescents.

**Figure 2:**
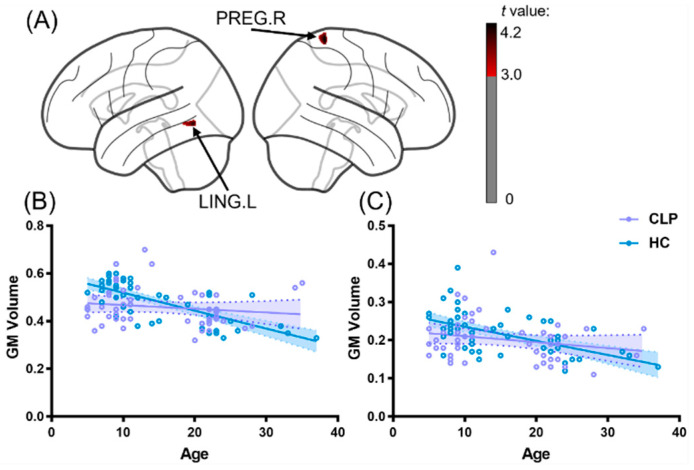
Specific gray matter (GM) regions showed significantly different correlations with age between CLP and HC. (**A**) Anatomical locations of brain regions whose GM volume exhibited significant interaction effects between group and age, including the left lingual cortex (LING.L) and the right precentral cortex (PREG,R); (**B**) age slopes of regional GM volume in LING.L in CLP and HC; (**C**) age slopes of regional GM volume in PREG.R in CLP and HC. Gender and total intracranial volume (TIV) were regressed out as covariates, and significance is set at cluster-wise Gaussian theory field (GRF)-corrected *p* < 0.05.

Using VBM analyses to detect group differences in GM volume between CLP and HC during development, we identified that, compared with HC, CLP participants had different GM volumetric alternating patterns over the course of development from adolescence to adulthood. In adolescent groups, CLP_Ado had a significantly decreased GM volume compared to HC_Ado in areas within the bilateral angular gyrus, left middle occipital gyrus and right parietal gyrus (cluster-wise GRF-corrected *p* < 0.05 with age, gender and TIV controlled) (Figure 1A). Meanwhile, for the adult groups, CLP_Adu had a significantly increased GM volume in the area within the left superior temporal and precentral gyrus and the right middle frontal cortex (Figure 1B). Furthermore, the GM volume of the two clusters showed significant interaction effects, located in the left lingual gyrus (LING.L) and the right precentral gyrus (PRENG.R) (Figure 2A). Relative to healthy controls, CLP participants exhibited a significantly smaller age-related GM volume in the LING.L (Figure 2B) and the PREG.R (Figure 2C). Detailed statistical results of the VBM analysis are summarized in Table 2.

### 3.3. The Differences in Cortical Thickness and Cortical Surface between CLP and Healthy Controls across Development

**Figure 3:**
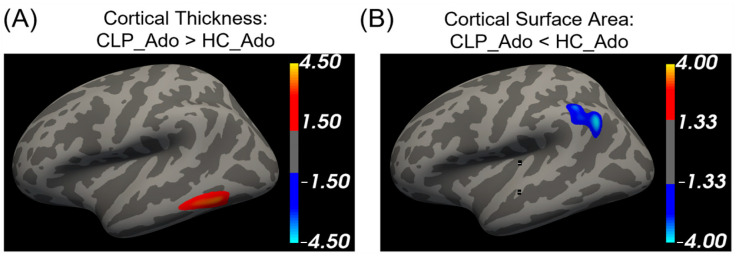
Significant group differences using surface-based morphometry analyses. (**A**) CLP adolescents have an increased cortical thickness compared to HC adolescents in the left inferior temporal cortex, which is colored red–yellow; (**B**) compared with HC adolescents, CLP adolescents have a decreased cortical surface area in the left inferior parietal cortex, which is colored blue. CLP_Adu = adults with cleft and palate (CLP), HC_Adu = healthy control adults, CLP_Ado = children and adolescents with CLP, HC_Ado = healthy children and adolescents. Gender and age were regressed out as covariates, and significance was set at cluster-wise *p* < 0.05 using Monte Carlo simulation with 5000 iterations.

**Figure 4:**
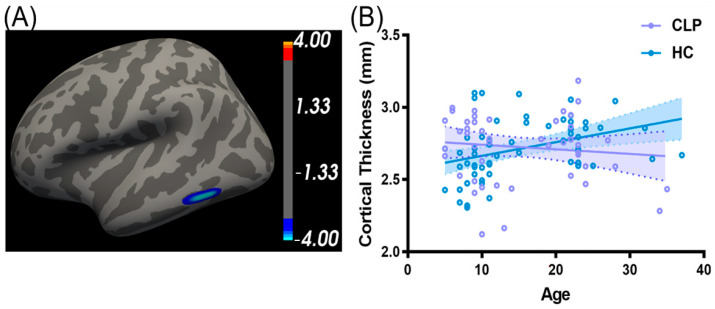
Cortical thickness of the left inferior temporal cortex exhibited a significant “group × age” interaction effect. (**A**) Significant interaction effect of “group × age” in the left inferior temporal cortex; (**B**) age slopes of regional mean cortical thickness in the left inferior temporal cortex in CLP and HC. Gender was regressed out as a covariate, and significance was set at cluster-wise *p* < 0.05 using Monte Carlo simulation with 5000 iterations.

Significant group differences in surface-based cortical structure were identified for adolescent groups. As presented in Figure 3, CLP_Ado had a significantly increased cortical thickness compared to HC_Ado in the area within the left inferior temporal cortex (ITG.L) and a decreased cortical surface area inside the left parietal cortex (cluster-wise *p* < 0.05, with age and gender controlled). No significant group differences were found between CLP_Adu and HC_Adu. In addition, the change in cortical thickness in the region of ITG.L with age was significantly different between CLP and HC participants (Figure 4A). The slope of the regression of cortical thickness against age was predominantly negative across the ITG.L in CLP, while it was positive in HC participants (Figure 4B).

**Figure 5:**
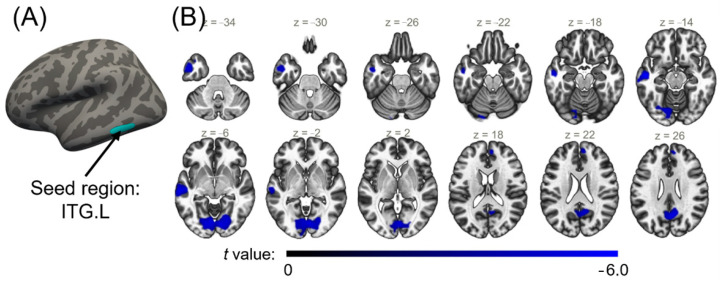
Decreased seed-based functional connectivity in CLP participants compared with HC. (**A**) The left inferior temporal cortex (ITG.L) was set as the seed, and the cortical thickness exhibited a significant “group × age” interaction effect.(**B**) Significantly decreased connectivity of CLP participants compared to HC in the functional connectivity analysis with ITG.L as the seed. Gender and age were regressed out as covariates, and significance was set at cluster-wise *p* < 0.05 with family-wise error (FWE) correction for multiple comparisons.

The ITG.L was identified as significantly interacting with age across groups. The cortical thickness of the ITG.L decreased with age in CLP, while it increased with age in healthy controls. We further investigated whether there existed significant differences in the functional connectivity of ITG.L between CLP and HC. Using seed-based functional connectivity with the ITG.L as the seed, we identified relatively decreased connectivity between IGT.L and specific regions, including the bilateral occipital fusiform gyrus, lingual gyrus, precuneus cortex, superior frontal gyrus and left middle temporal cortex, in CLP compared to HC (cluster-wise few-corrected *p* < 0.05, with age and gender being controlled) (Figure 5). Detailed statistical results of the SBM analysis are summarized in Table 3.

### 3.4. Mediation Effect of Cortical Structure between Age and Articulation Function in CLP

**Figure 6:**
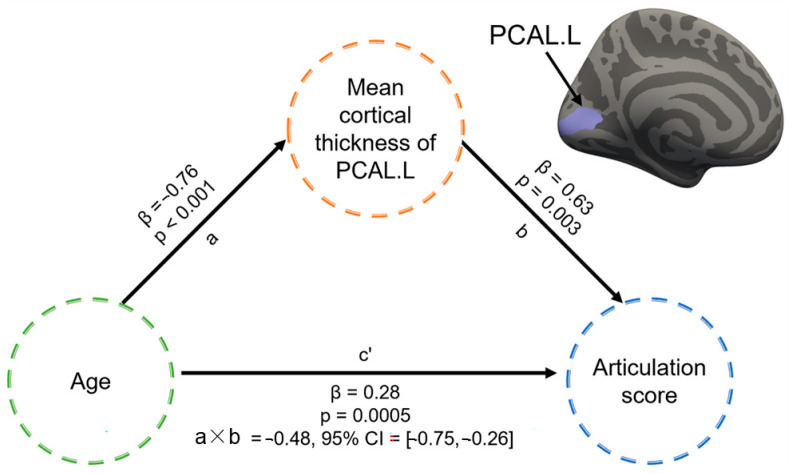
Significant mediation effect of the left pericalcarine cortex (PCAL.L) between age and articulation score in CLP. Path a: the relationship between age and mean cortical thickness of the PCAL.L; Path b: the relationship between mean cortical thickness of PCAL.L and the articulation performance; Path c’: the relationship between age and the measures of articulation function; Path a × b represents an indirect path, namely, the authors declare a relationship between age and articulation function, which was mediated by the mean cortical thickness of the PCAL.L. A significant mediation effect is deemed when the 95% bootstrapping confidence interval based on 10,000 bootstrap samples does not contain zero.

Considering the association between age and articulation function (r = 0.28, *p* = 0.058, with gender controlled), mediation analysis was performed to assess the association among cortical structure, age and articulation function. Among the cortical structural features of regions exhibiting significant group differences or interaction effects and regions parcellated across the Desikan–Killiany Atlas, we identified that the significant correlation between age and CLP participants’ articulation test scores was significantly mediated by the mean cortical thickness of the left pericalcarine cortex (PCAL.L), controlling for gender (Figure 6). The analysis suggested the existence of a partial mediation effect (a × b = −0.48, 95% confidence interval (−0.75, −0.26)) of the cortical thickness of the PCAL.L, with a direct effect (path c’: β = 0.28, *p* = 0.0005) of age on articulation function.

## 4. Discussion

Using structural and resting-state functional MRI data, we identified an age-related abnormal pattern of cortical development in CLP. From the development from childhood to adulthood, we identified that patients with CLP exhibited distinct brain structural development compared with age-matched healthy controls, especially for the cortical thickness of the left inferior temporal cortex and the GM volume of the left lingual gyrus and right precentral gyrus. Using the left inferior temporal cortex as the seed, we further revealed that CLP patients had significantly decreased seed-based functional connectivity to specific regions compared to healthy participants, including the bilateral occipital fusiform gyrus, lingual gyrus, precuneus cortex, superior frontal gyrus and left middle temporal cortex. Indeed, we found that the effect of age on articulation function was partially mediated by the cortical thickness of the left pericalcarine cortex in CLP.

### 4.1. Specific Structural Changes in CLP between Different Age Durations, Adolescence vs. Adult

Using the VBM analysis, we found that the group differences in GM volume between CLP and HC had a distinct spatial distribution during the development from adolescence to adulthood. In the comparison of adolescent groups, CLP_Ado displayed a significantly decreased GM volume in regions within the bilateral angular gyrus, left middle occipital gurus, right superior parietal gyrus and right postcentral gyrus compared to HC_Ado. Meanwhile, in the adult groups, significantly increased GM volumes within the left superior temporal gyrus, left precentral gyrus and right middle frontal gyrus were observed in CLP_Adu compared to HC_Adu. These significant brain regions are in accordance with the cortical structure and brain functions in previous CLP and language process studies [14,25,30]. The angular gyrus is considered the language hub for modulation [31], primarily involved in language comprehension and phonological production [32,33]. The middle frontal gyrus is a well-known secondary language area [34] which engages in expressive language processes such as semantics [35], syntax and grammar [36], verbal fluency [37] and verbal working memory [38]. The postcentral gyrus is associated with phonetic articulatory planning and execution [39]. These results demonstrate that GM developmental patterns between CLP adolescents and CLP adults are radically different. CLP adolescents showed decreased GM volume in regions mainly involved in language processing and visual learning functions [40], suggesting a weaker language expression ability of CLP adolescents. Articulation disorders are often associated with the reduced expressive vocabulary of CLP children [41]. Moreover, CLP adults showed an increased GM volume in the regions involved in repeated vowel perception and production [42], speech production [43] and the reorienting of attention [44]. These results demonstrate that CLP adults require greater muscular control and prosody control in their speech process, which might help them improve their articulation function.

In the analysis of SBM, we identified that CLP also had different cortical structural alternations compared with healthy controls across different age durations. A significantly decreased cortical surface area in the left parietal cortex and a significantly increased cortical thickness in the left inferior temporal cortex were observed in CLP_Ado when compared with HC_Ado, while no significant group differences in cortical structure between adult groups was identified using SBM analysis. Previous studies also reported an increased cortical thickness of the left interior temporal cortex [45,46], which is mainly involved in the processing of verbal fluency [47]. The reduced surface area of the left temporal cortex in CLP adolescents may be associated with verbal fluency [41] and receptive vocabulary [5] problems. There are cortical and subcortical structures in the left parietal cortex that help with language processing [48]. We speculate that this was caused by CLP adolescents’ complicated semantic processing capabilities [41] and that the increased cortical thickness was a result of compensatory measures for the condition.

### 4.2. Cortical Structural Changes in CLP Participants during Development

We performed multiple regression modeling to investigate if there was a distinct cortical developmental pattern in CLP participants during the development from childhood to adulthood. Significantly different age slopes against regional GM volume in the LING.L and the PREG.R were identified for CLP. These significant regions are completely different from the previous studies on language function and cortical structural changes in normal children during development, demonstrating the development specificity of cortical structures in CLP patients. The LING is involved in the visual analysis of word forms, particularly in word processing [49,50,51]. The PREG gyrus is a motor area that receives proprioceptive impulses from muscles and joints and is involved in the articulatory processes [52,53]. Our findings suggest that CLP patients with articulation disorder may use more neural pathways in the visual-function-related area to compensate for the language and speech dysfunction. These results demonstrate an association between the effect of articulation disorder and the cortical structure of CLP patients during development. This evidence confirms the influence of articulation disorder and reveals the potential neural mechanism behind this influence: visual and motor functions compensate for some of the deficits in the articulation function of CLP patients during development.

Significantly different age slopes against regional cortical thickness in the left inferior temporal cortex were identified for CLP. An interesting phenomenon was that our results indicated a decreased cortical surface area in the left inferior temporal cortex and an increased cortical thickness in the left inferior parietal cortex in CLP. The abnormal function and structure of these language-related cortexes were reported to be associated with verbal fluency [47] and language processing [48] dysfunction. Decreased seed-based functional connectivity of ITG.L was observed, mainly connected with regions located in the bilateral precuneus cortex, superior frontal gyrus and left temporal gyrus, which are usually regarded as essential compartments of the default mode network and are anatomically located closer to the Wernick’s area, one of the most critical language-related areas [54,55,56].

### 4.3. Mediation Effect of the Left Pericalcarine Cortex between Age and Speech Performance of CLP

In contrast with previous studies [8,9,10,13], we sought to evaluate the association between cortical structure development and articulation function along with age from childhood to adulthood. Using mediation analysis, we identified that the effect of age on articulation function in CLP individuals was partially moderated by the regional cortical thickness within the PACL.L (Figure 6). The PCAL.L is closely related to visual function and serves as the primary visual processing cortex [57]. According to previous studies, language-related processing tasks include word recognition, verbal fluency (letter and category), grapheme processing, the encoding and maintenance of linguistic information, sentence structure building and/or retrieval and/or the retrieval of word meanings from long-term memory [58,59,60]. Due to CLP being a congenital deformity, there may be deficits in higher cortical cognitive functions and semantic processing in CLP. We presumed that the PCAL.L undergoes partial compensation in semantic processing in CLP. The left occipital region may be involved in the language function during early language acquisition. Therefore, the PCAL.L experiences partial compensation in semantic processing. Consequently, the associations we found among age, the cortical thickness of the PCAL.L and articulation function need to be investigated in further studies on speech disorders in the CLP population, which might help to provide more information on assessing appropriate treatment strategies (e.g., speech rehabilitation) and thus to improve functional speech outcomes in CLP patients. Overall, these findings support a new hypothesis that the association between age and articulation function in CLP patients is regulated by cognitive procedures, including visual information reception and processing.

## 5. Limitations

There are several limitations in the current study. Our study had a small sample size, and the language function assessment scale was relatively homogeneous. For future research, more clinical variables that may influence speech disorders in CLP should be considered, including age at surgery, type of cleft palate, speech training, palatopharyngeal closure and home language environment, which should be controlled for in future research.

## 6. Conclusions

Our results revealed the distinct distribution of abnormal cortical structures in adolescent and adult CLP patients. We also found that the developmental pattern of cortical structures in CLP patients differed from the pattern in healthy controls. Mediation analysis identified the cortical thickness of the left pericalcarine cortex as the mediator between age and articulation function. In conclusion, our results demonstrate an abnormal developmental pattern of cortical structures in CLP patients, which is directly related to their articulation disorders.

There are several limitations in the current study. Our study had a small sample size, and the language function assessment scale was relatively homogeneous. For future research, more clinical variables that may influence speech disorders in CLP should be considered, including age at surgery, type of cleft palate, speech training, palatopharyngeal closure and home language environment, which should be controlled for in future research. In order to further evaluate the distribution of brain regions with distinctive developmental patterns in the adolescent to adult phases of CLP patients, as reported in our findings, future studies should include a broader age range of CLP participants. In addition, given the substantial link between brain structure and function, the follow-up study will collect multi-modal brain neuroimaging data in order to evaluate more characteristic modification patterns of structural and functional brain regions in the CLP population.

## Figures and Tables

**Table 1:** Demographic and clinical characteristics of participants enrolled in this study.

	CLP	HC	*T/X* ^2^	*p*
	(*n* = 49)	(*n* = 59)		
Adolescents (n = 63)	CLP_Ado = 26	HC_Ado = 37		
Age, years *^a^*	8.88 (2.39) [5–14]	9.89 (2.72) [5–16]	−1.52	0.133
Gender (Female/Male)*^b^*	8/18 (44.4%)	10/27 (55.6%)	0.11	0.746
Articulation test score	44.83 (25.73) [0–93]	--	--	*--*
Adults (n = 45)	CLP_Adu = 23	HC_Adu = 22		
Age, years (range) *^a^*	23.35 (4.16) [18–35]	24.41 (4.38) [19–37]	−0.83	0.409
Gender (Female/Male) *^b^*	6/17 (30.0%)	14/8 (70%)	6.42	0.011
Articulation test score	51.00 (23.57) [13–93]	--	--	*--*
Total (n = 108)	CLP = 49	HC = 59		
Age, years (range) *^a^*	15.67 (8.01) [5–35]	15.31 (7.85) [5–37]	0.24	0.810
Gender (Female/Male) *^b^*	14/35 (36.8%)	24/35 (63.2)	1.72	0.190
Articulation test score	46.82 (23.92) [0–93]			

Numerical data are presented as: mean (standard deviation) [range]. CLP_Adu = adults with cleft and palate (CLP), HC_Adu = healthy control adults, CLP_Ado = children and adolescents with CLP, HC_Ado = healthy children and adolescents. *^a^* Independent *t*-test. *^b^* Chi-square test.

**Table 2:** Group differences in gray matter (GM) intensity based on volume-based morphometry analyses.

	No. Cluster	Cluster Size	Peak MNI Coordinates			Peak *t* Value	Anatomic Locations Based on AAL Atlas
			x	y	z		
Significant group differences ^a^							
Ado: CLP < HC	1	107	31.25	48.75	1.76	−3.31	Left angular gyrus
	2	237	31.25	48.75	1.77	−3.73	Right angular gyrus
	3	283	31.25	48.75	1.78	−4.01	Left middle occipital gyrus
	4	136	31.25	48.75	1.79	−3.69	Right angular gyrus
	5	228	31.25	48.75	1.8	−3.92	Right superior parietal gyrus
	6	175	31.25	48.75	1.81	−4.12	Right postcentral gyrus
Adu: CLP > HC	1	125	31.25	48.75	1.82	3.53	Left superior temporal gyrus
	2	113	31.25	48.75	1.83	3.94	Right middle frontal gyrus
	3	121	31.25	48.75	1.84	4.14	Left precentral gyrus
Interaction: Group × Age ^b^							
CLP > HC	1	112	−21.75	−61.25	−6.25	4.02	Left lingual gyrus
	2	260	36.25	−42.25	72.75	4.19	Right precentral gyrus

No. cluster = number of clusters, MNI = Montreal Neurological Institute space, Ado = adolescents, Adu = adults. Significance level was set at cluster-wise *p* < 0.05 with Gaussian random field (GRF) correction. ^a^ Independent *t*-test with age, gender and TIV controlled. ^b^ Statistical analysis was conducted using a general linear model. Gender and TIV were controlled as covariances. Significant differences between the group–age slopes were regarded as representing significant interactions between group and age.

**Table 3:** Statistical results using surface-based morphometry analyses.

	No. Cluster	Cluster Size (mm^2^)	Peak MNI305 Coordinates			Peak z Value	Anatomic Locations Based on AAL Atlas
			x	y	z		
Significant group differences ^a^							
Ado: CLP < HC (cortical thickness)	1	644.5	−54.2	−57.6	−12.2	3.68	Left inferior temporal cortex
Ado: CLP > HC (surface area)	1	845.36	−50.5	−59.4	28.9	−3.89	Left inferior parietal cortex
Interaction: Group × Age (cortical thickness) ^b^							
CLP < HC	1	374.58	−55.4	−52.2	−14.5	−4.36	Left inferior temporal cortex

No. cluster = number of clusters, MNI = Montreal Neurological Institute space, Ado = adolescents, Adu = adults. Significance level was set at cluster-wise *p* < 0.05 with family-wise error (FWE) correction. ^a^ Independent *t*-test with age and gender controlled. ^b^ Statistical analysis was conducted using a general linear model. Gender was controlled as a covariance. Significant differences between the group–age slopes were regarded as representing significant interactions between group and age.

## Data Availability

The data that support the findings of this study are available from the corresponding author on reasonable request.

## Supplementary Materials

The following are available online at https://www.mdpi.com/article/10.3390/brainsci13040550/s1, Table S1: The inclusion and exclusion criteria of the CLP participants, Table S2: The inclusion and exclusion criteria of the healthy participants.
