# Supplementary material for: Articulation-Function-Associated Cortical Developmental Changes in Patients with Cleft Lip and Palate
Source: Brain Sci. 2023 Mar 25;13(4):550. doi: 10.3390/brainsci13040550 (PMC10136718; doi:10.3390/brainsci13040550)
Supplement: Supplementary file 1 [file brainsci-13-00550-s001.zip › brainsci-2263778-supplementary.pdf]

The inclusion and exclusion criteria of the participants

Table S1 The inclusion and exclusion criteria of the CLP participants

**The inclusion criteria of CLP participants**		
All patients with cleft lip and palate, and have good recovery of palatopharyngeal function after palatoplasty surgery more than 1 year.	□1 Yes	□0 No
The age of CLP adolescents is between 5 and 16 years, and the age of CLP adults is between 18 and 40 years	□1 Yes	□0 No
The native language is Chinese	□1 Yes	□0 No
Normal intelligence quotient (IQ)≥90,Wechsler Intelligence Scale	□1 Yes	□0 No
Right-handed	□1 Yes	□0 No
Free of neurological disease ,psychiatric disorders and contraindications of fMRI	□1 Yes	□0 No
CLP subjects had two or more typical characteristics of articulation disorder, such as hypernasality, nasal emission, weak or absent of consonants and compensatory misarticulations.	□1 Yes	□0 No
CLP subjects with articulation disorder had score ≤70 points in articulation test.(Chinese language clear degree list)	□1 Yes	□0 No
All subjects had not received speech training.	□1 Yes	□0 No
**The exclusion criteria of CLP participants**		
neurological or psychiatric disorders and contraindications of fMRI	□1 Yes	□0 No
Systematic medication and psychotherapy within 1 year prior to enrollment	□1 Yes	□0 No
Inability to cooperate with the relevant assessment and examination	□1 Yes	□0 No
Refused to participate in fMRI experiment	□1 Yes	□0 No

Table S2 The inclusion and exclusion criteria of the healthy participants

**The inclusion criteria of healthy participants**		
The age of healthy children and adolescents between 5 and 16 years, and the age of healthy adults is between 18 and 40 years.	□1 Yes	□0 No
The native language is Chinese	□1 Yes	□0 No
Normal intelligence quotient：Normal intelligence by clinical screening	□1 Yes	□0 No
Right-handed	□1 Yes	□0 No
Free of neurological disease , psychiatric disorders and contraindications of fMRI	□1 Yes	□0 No
clinically screened for normal articulation function by a speech therapist	□1 Yes	□0 No
**The exclusion criteria of Healthy participants**		
neurological or psychiatric disorders and contraindications of fMRI	□1 Yes	□0 No
Systematic medication and psychotherapy within 1 year prior to enrollment	□1 Yes	□0 No
Inability to cooperate with the relevant assessment and examination	□1 Yes	□0 No
Refused to participate in fMRI experiment	□1 Yes	□0 No
